# Enterococcus: Understanding Their Resistance Mechanisms, Therapeutic Challenges, and Emerging Threats

**DOI:** 10.7759/cureus.79628

**Published:** 2025-02-25

**Authors:** Sourish Hota, Satish R Patil, Priyanka M Mane

**Affiliations:** 1 Department of Microbiology, Krishna Institute of Medical Sciences, Krishna Vishwa Vidyapeeth (KVV), Karad, IND

**Keywords:** enterococcus faecalis (e. faecalis), enterococcus faecium, group d streptococcus, healthcare-associated infection (hai), linezolid (lzd), vancomycin-resistant enterococcus (vre), vitek 2

## Abstract

The *Enterococcus* species originates as non-harmful bacteria indigenous to human intestines but has transformed into severe hospital-acquired pathogens due to antimicrobial resistance (AMR). The clinical species *Enterococcus faecalis* and *Enterococcus faecium* create the most relevant infections because they appear in urinary tract infections, bloodstream infections, endocarditis, and wound infections. *Enterococcus* species demonstrate multiple antibiotic class resistance and resistance determinant acquisition properties that make treatment difficult for medical professionals. Vancomycin-resistant enterococci (VRE) together with high-level aminoglycoside-resistant strains and resistance to both linezolid and daptomycin have exhausted available treatment options. The review investigates the development process of *Enterococcus* infections by examining virulence characteristics, which involve biofilm production and defense mechanisms against the immune response and transmission of resistance genes. A thorough investigation of medical publications used Google Scholar along with PubMed and ScienceDirect and Medical Subject Headings (MeSH) as appropriate search terms. The traditional classification of *Enterococcus* species from historical context to modern epidemiology and pathogenesis and available treatment and test approaches are explained in this review. This section examines two categories of resistance together with their mechanisms of action with a specific focus on vancomycin resistance produced by van gene clusters as well as its prevalence trends. An examination of how horizontal gene transfer functions in transferring resistance throughout healthcare facilities is included. The paper investigates the different symptoms of enterococcal infections together with diagnostic obstacles and treatment modalities. Drug-resistant *Enterococcus* infections continue to increase internationally, so healthcare professionals need new therapeutic methods, better antimicrobial policies, and stronger infection prevention measures. The examination surveys *Enterococcus* infections through an extensive evaluation of developing resistance patterns combined with emerging intervention requirements.

## Introduction and background

Once thought innocuous commensals, the species of *Enterococcus* have risen to become notable nosocomial pathogens responsible for a vast armamentarium of healthcare-associated infections. Gram-positive, facultatively anaerobic, and extremely tolerant bacteria colonizing soils, fresh water, products intended for consumption, and other substrates by means of wastewater, they become highly opportunistic in their expression [1]. Although they are normally part of the human and animal gut microbiota, *Enterococcus* species have increasingly been linked to severe infections, including urinary tract infections (UTIs), bloodstream infections (BSIs), endocarditis, and wound infections. Their persistence in hospital environments, coupled with their intrinsic and acquired resistance mechanisms, makes them a significant challenge in clinical settings [2].

## Review

Search strategy

**Figure 1:**
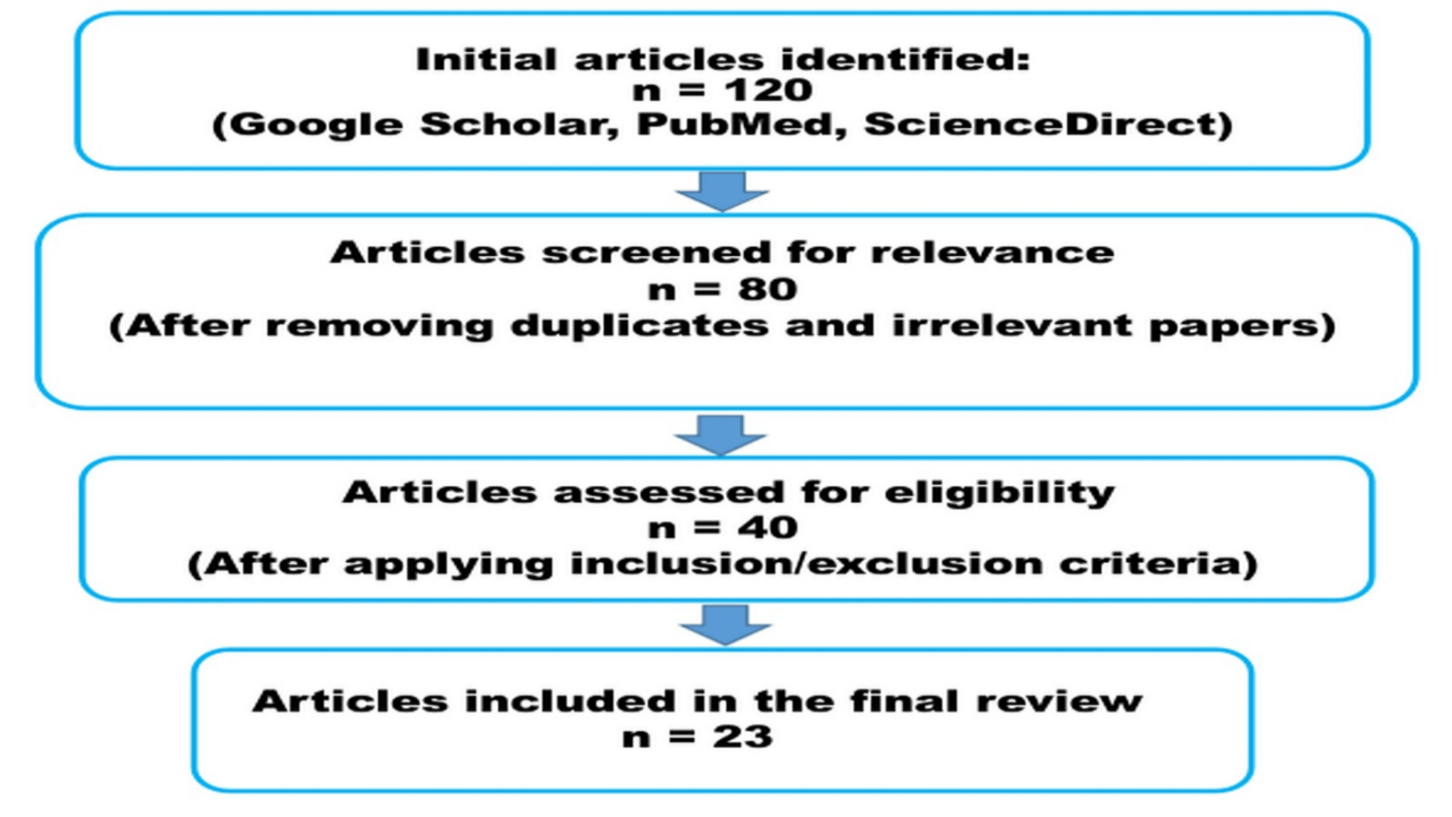
Article Selection and Screening Process n: number of articles

A thorough examination of literature sources including Google Scholar, PubMed, and ScienceDirect served as the foundation for creating this review article about *Enterococcus*. The search covered publications from 2017 to 2025 and utilized Medical Subject Headings (MeSH) terms such as “history of *Enterococcus*,” “taxonomy of *Enterococcus*,” “*Enterococcus* infections,” “virulence and pathogenicity of *Enterococcus*,” “laboratory diagnosis of *Enterococcus* infections,” “treatment of *Enterococcus* infections,” “antimicrobial resistance in *Enterococcus*,” and “vancomycin-resistant *Enterococcus* (VRE).” Research publications that did not meet the inclusion criteria for being written in English or concerning *Enterococcus *pathogenesis, diagnosis, treatment, or antimicrobial resistance (AMR) were excluded along with studies that lacked full-text access. The initial database review produced 120 results for analysis. A total of 80 articles remained after duplicate papers and irrelevant content were removed from the initial 120 papers identified. The final review incorporated 23 articles that passed through identification selection and inclusion and exclusion criterion evaluation processes starting from 40 initially assessed articles. This review constructs its evidence-based discussion after analyzing the entire literature pool of chosen sources (Figure 1).

Brief scenario

Among over 30 known *Enterococcus* species, the most clinically relevant are *Enterococcus faecalis* and *Enterococcus faecium*, which cause the majority of human infections. Recently recognized clinically relevant *Enterococcus* species include *Enterococcus gallinarum*, *Enterococcus casseliflavus*, *Enterococcus raffinosus*, *Enterococcus hirae*, *Enterococcus durans*, and *Enterococcus mundtii*, which are increasingly found in infections, particularly in immunocompromised patients. They are highly adapted, tolerating adverse conditions, including high temperature (up to 60°C for 30 minutes), high pH (9.6), and hyperosmotic conditions (6.5% NaCl, 40% bile salts) [2,3]. The fact that they can colonize and survive on medical devices, such as catheters and ventilators, also provides additional evidence for their nosocomial infection potential. The most alarming characteristic of *Enterococcus* infections is their high degree of AMR, which severely limits available interventions. *Enterococcus* species possess intrinsic resistance to most antibiotics, such as cephalosporins, low-level aminoglycosides, and clindamycin. Much more foreboding, however, is their ability to develop high-level resistance to the glycopeptides (vancomycin-resistant enterococci (VRE)), high-level aminoglycosides, and oxazolidinones (linezolid resistance). VRE, particularly in the species *E. faecium*, has raised significant therapeutic issues, resulting in increased morbidity, mortality, and healthcare costs. Furthermore, the transmission of resistance by mobile genetic determinants, such as plasmids and transposons, exacerbates the crisis due to the extensive spread of resistance in a healthcare environment [3].

Treatment of *Enterococcus* infection has increasingly become more complicated due to the robust antibiotic resistance to these pathogenic micro-organisms. While the antibiotic treatment of *E. faecalis* infections using ampicillin has proven effective, *E. faecium* remains much more difficult to treat, hence the use by medical practitioners of advanced drugs, which include tigecycline, daptomycin, and linezolid. The resistance has become almost immediate to any available treatment options, thus the increased urgency in the development of new alternatives. The beta-lactam-aminoglycoside synergy works best for severe diseases, but due to the undefined high prevalence of resistance to aminoglycosides, their use in combination therapies is less likely to be achieved. New antimicrobials, indeed, along with other treatment options are immediately needed for this ever-emerging group, which has increasingly been found to be demonstrating resistance to drugs [4].

This review aims to provide a comprehensive understanding of *Enterococcus* infections, focusing on their virulence factors, AMR mechanisms, and current therapeutic challenges. By exploring the evolving resistance patterns and available treatment options, we seek to highlight the growing concern of antibiotic-resistant *Enterococcus* and the need for innovative solutions in managing these infections.

Pathogenesis and virulence factors

Nosocomial enterococcal infections stand as a concerning healthcare threat because these bacteria demonstrate successful survival within hospital facilities while binding to medical instruments and creating protective biofilms against immune system responses and antibiotic exposure. The high prevalence of *E. faecium* BSIs reaches 74%, thus underscoring both the necessity for rigorous infection prevention practices and new treatment approaches because of the patients' unfavorable prognosis. The pathogenic mechanisms of *Enterococcus* species depend on two main surface proteins: Esp, which helps form biofilms and causes UTIs, and *EfaA* and *Ace*, which lead to infective endocarditis and osteomyelitis. Medical devices become infected by enterococcal biofilms, which improve their capability to fight off immune defenses and antibiotic therapies. Through *Esp* and cytolysin, enterococci disable the activity of neutrophils and macrophages to evade immune response. Cytolysin and two proteases as well as gelatinase (GelE) and lipases work together to enable tissue damage and biofilm formation. *Enterococcus* can produce beta-lactamase uncommonly yet enables complete penicillin resistance when it occurs. Sex pheromones named *Cob* and *Ccf* aid the process of horizontal gene transfer, thus making it possible to disseminate antibiotic resistance features together with virulence factors. The pathogenic strategies of enterococci help them thrive in persistent infections and evade treatment while creating problems for medical staff who need to develop new therapeutic methods [5].

Clinical manifestations

The presence of enterococcal infections occurs across different clinical situations leading to important cases of both mortality and morbidity. The occurrence of enterococci infections stands at a rate of 9.8% among all cases. Enterococcal endocarditis leads to sub-acute heart failure symptoms, which cause death in 9%-15% of cases more than other types of infective endocarditis. Meningitis is considered a rare infection (4%) that develops from *E. gallinarum* and *E. casseliflavus* when patients have diabetes or renal failure or exhibit immunosuppressant behavior. Shunt devices contribute to the development of nosocomial meningitis, which kills 20% of patients. Cirrhosis and dialysis patients develop high mortality rates and septicemia from enterococci-caused peritonitis (intra-abdominal infections) and pelvic and soft tissue infections. Post-surgical infections affecting the salpingitis and endometritis and post-cesarean abscesses are commonly caused by enterococci while endophthalmitis following cataract surgery from this pathogen leads to inferior visual results than *Staphylococcus* infections [6].

Laboratory diagnosis of enterococcal infections

The diagnosis of enterococcal infections starts with microbiological analysis and proceeds through biochemical assessment alongside antibacterial resistance testing. The granulose bacteria manifest as chains or pairs of cocci, which tolerate 6.5% salt concentration and grow at 45°C. Biochemical tests like bile esculin hydrolysis together with pyrrolidonyl-arylamidase (PYR) testing aid identification processes [7]. Rapid species identification is possible through the combination of polymerase chain reaction (PCR) and matrix-assisted laser desorption ionization time-of-flight mass spectrometry (MALDI-TOF MS) testing methods. The VITEK 2 system (bioMerieux, Marcy-l'Étoile, France) operates as a widely employed automated microbiology analyzer that delivers dual identification and susceptibility testing (can also be performed by the Kirby-Bauer disk diffusion method) while improving accuracy and efficiency. The breakpoints for antibiotic susceptibility testing of *Enterococcus* have been elicited in Table 1 [8].

**Table 1:** Breakpoints for Antibiotic Susceptibility Testing of Enterococcus (Disk Diffusion & MIC Interpretation) by CLSI [8] S: sensitive; I: intermediate; R: resistant; SDD: susceptible-dose-dependent; KB-DD: Kirby-Bauer disk diffusion; MIC: minimum inhibitory concentration; µg: microgram; mm: millimeter; *E. faecalis*: *Enterococcus faecalis*

Antibiotics	Disk content	KB-DD zone diameter interpretation (mm)			MIC interpretation (µg/mL)			
		S	I	R	S	SDD	I	R
Penicillin	10 units	≥15	-	≤14	≤8	-	-	≥16
Ampicillin	10 µg	≥17	-	≤16	≤8	-	-	≥16
Teicoplanin	30 µg	≥14	11-13	≤10	≤8	-	16	≥32
Vancomycin	30 µg	≥17	15-16	≤14	≤4	-	8-16	≥32
Daptomycin (for *E. faecalis* only)	-	-	-	-	-	≤4	-	≥8
Tetracycline	30 µg	≥19	15-18	≤14	≤4	-	8	≥16
Ciprofloxacin	5 µg	≥21	16-20	≤15	≤1	-	2	≥4
Nitrofurantoin	300 µg	≥17	15-16	≤14	≤32	-	64	≥128
Fosfomycin	200 µg	≥16	13-15	≤12	≤64	-	128	≥256
Quinipristin-dalfopristin	15 µg	≥19	16-18	≤15	≤1	-	2	≥4
Linezolid	30 µg	≥23	21-22	≤20	≤2	-	4	≥8

AMR in *Enterococcus*

Intrinsic Resistance

*Enterococcus* exhibits intrinsic resistance to multiple antibiotics due to their structural and metabolic features. Beta-lactam antibiotic resistance occurs through penicillin-binding protein 5 (PBP5) because the protein shows lower penicillin affinity due to different structural features than other PBPs. PBP5 stands apart from essential cell wall synthesis PBPs, PBP1 and PBP2, because its active site structure differs and its beta-lactam attachment efficiency is low, which enables its survival during antibiotic treatment. The structural modification works as an intrinsic resistance factor in *Enterococcus* spp. The emergence of high-level beta-lactam resistance develops from the simultaneous occurrence of PBP5 overexpression together with specific mutations that cause resistance levels spanning 16-64 µg/mL [9]. Aminoglycoside resistance occurs because *Enterococcus* species lack necessary electron transport systems, which limits their uptake capacity to create low-level resistance (62-500 µg/mL minimum inhibitory concentration (MIC)). The therapeutic outcome for endocarditis-specific severe infections benefits from beta-lactam and glycopeptide combination therapy with aminoglycosides due to their limited individual effectiveness [9,10].

Acquired Resistance

Multidrug-resistant enterococci form because these bacteria alternatively develop resistance through genetic mutations and obtain resistant genes by horizontal transfer [11]. The major clinical threat of VRE exists because of the presence of van gene clusters. Plasmids carrying the VanA phenotype provide bacteria with high-level resistance to vancomycin together with teicoplanin whereas VanB found frequently in bacterial chromosomes gives variable vancomycin resistance but keeps teicoplanin sensitivity [11,12]. Other variants that exist as VanC, VanD, VanE, and VanG types provide reduced resistance compared to conventional strains. High-level aminoglycoside resistance (HLAR) becomes active when MIC tests show values higher than 2,000 µg/mL, which makes aminoglycosides unable to be effective during combination therapy. AMEs along with ribosomal mutations act as resistance mediators, which either destroy antibiotics or modify the drug-binding locations on ribosomes. Different resistance genes including *aph(2')-Ic*, *aph(2”)-Id*, and *aph(2”)-Ib* determine specific resistance patterns toward various aminoglycoside drug groups. The 23S rRNA gene develops mutations that create reduced drug binding sites to form linezolid resistance [12,13]. The *optrA* gene controls an efflux pump operation, yet the *cfr* gene modifies 23S rRNA by methylation making antibiotics unable to bind. The modification of membrane structure alongside potential changes leads to decreased bactericidal effects of daptomycin. Medical treatment becomes challenging because of these antibiotic resistance mechanisms that may require healthcare providers to switch to therapeutic options such as tigecycline or fosfomycin [14]. Various AMR mechanisms in *Enterococcus* are discussed in Table 2.

**Table 2:** Antimicrobial Resistance Mechanisms in Enterococcus, Patterns, and Genetic Determinants AAC: aminoglycoside acetyltransferase; ANT: aminoglycoside nucleotidyltransferase; APH: aminoglycoside phosphotransferases; cfr: chloramphenicol-florfenicol resistance; mprF: multiple peptide resistance factor; van: vancomycin resistance gene; PBP: penicillin-binding protein; bla: beta-lactamase; gyr: DNA gyrase; parC: topoisomerase IV; cat: chloramphenicol acetyltransferase; erm: erythromycin resistance methylase; tet: tetracycline resistance protein; optr: oxazolidinone phenicol resistance gene; poxt: phenicol-oxazolidinone-tetracycline resistance gene; fos: fosfomycin-resistance-related genes; murA: UDP-N-acetylglucosamine enolpyruvyl transferase; MLS: macrolide, lincosamide, and streptogramin B

Resistance pattern	Antibiotics affected	Mechanism of resistance	Key determinants
High-level resistance to aminoglycosides [11-16]	Gentamicin, kanamycin, streptomycin	1. Enzymatic modification (aminoglycoside-modifying enzymes). 2. Alteration of the target (decreased ribosomal binding)	AAC(6’)-Ie + APH(2”)-Ia AAC(6’)-Ii APH(3’)-IIIa ANT(3’)-Ia ANT(4’) ANT(6’)-Ia
Resistance to glycopeptides [17]	Vancomycin, teicoplanin	Alteration of the target (modification of the peptidoglycan biosynthetic pathway)	VanA, VanB, VanC (VanC1, VanC2, VanC3), VanD, VanE, VanG
Resistance to beta-lactams [18]	Penicillin, ampicillin	1. Alteration of the target (modification of PBPs). 2. Enzymatic degradation (beta-lactamase production)	pbp5, blaZ
Resistance to quinolones [19]	Ciprofloxacin, levofloxacin	Alteration of the target (mutations in DNA gyrase and topoisomerase IV)	gyrA, parC
Resistance to chloramphenicol [13]	Chloramphenicol	Enzymatic inactivation (chloramphenicol acetyltransferase)	cat
Resistance to MLS group [15]	Macrolides (erythromycin), lincosamides (clindamycin), streptogramin B	Enzymatic modification (methylation of ribosomal targets)	erm(B)
Resistance to tetracyclines [16]	Tetracycline, doxycycline	Efflux pump-mediated resistance, ribosomal protection proteins	tet(M), tet(L), tet(O)
Resistance to oxazolidinones [14]	Linezolid	1. Target site modification (mutations in 23S rRNA and ribosomal proteins). 2. Enzymatic inactivation (cfr-mediated methylation)	cfr, optrA, poxtA
Resistance to daptomycin [17]	Daptomycin	Alteration of the cell membrane (increased cell wall thickness, mutations in *mprF)*	mprF
Resistance to fosfomycin [20,21]	Fosfomycin	Enzymatic inactivation (fosfomycin-modifying enzymes), mutation in transporters	fosB, murA

Horizontal gene transfer in resistance

The main mechanism of enterococci resistance transmission occurs through horizontal gene transfer, including conjugation, transposition, and transformation. Hospital staff face serious risks from the plasmid-mediated transfer mechanism, which spreads both vancomycin resistance genes and aminoglycoside genes. The sexual attraction mechanism through sex pheromones allows specific *E. faecalis* strains to improve conjugation efficiency. Tn1546 represents a transposon that enters bacterial chromosomal material to promote long-term survival because of its VanA resistance functions. The acquisition through the transformation of resistance genes from environmental DNA makes enterococci more adaptable [15].

Historical background of VRE

At the end of the 19th century, scientists first identified *Enterococcus* pathogens by tracing *E. faecalis* infections, which caused septicemia together with endocarditis in human patients. The medical significance of *Enterococcus* infections increased as their occurrence gradually rose over time. Research on hospital-acquired infections led to the first documented publication in 1976, and high-level gentamicin resistance (HLGR) was first confirmed by scientists in France in 1979. Medical science discovered VRE for the first time in England in 1988 before France and the United States confirmed the phenomenon in the early 1990s. Between the late 1990s and 2000 through 2006, the number of enterococci infections in America rose to thousands while VRE cases expanded to double their original numbers. The lack of research about VRE incidence in Indian territories becomes evident, yet future studies paired with infection control strategies should be established as a priority [16].

Scientific literature has shown VRE on the rise in India. Of 3,683 enterococci isolates examined between 2000 and 2022, 12.4% were vancomycin-resistant. The prevalence increased from 4.8% between 2000 and 2010 to 14.1% between 2011 and 2020. A descriptive study in 2017-2018 in a western Indian tertiary care center had a prevalence of 11.13% VRE among *Enterococcus* isolates. Among all the VRE isolates, 49.06% were from urine samples, and 32.08% were isolated through investigations by blood cultures [17-19].

Animal farming dependent on antibiotic usage has been instrumental in developing the VRE infection. Animals across Europe developed a high prevalence of VRE because avoparcin, which resembles vancomycin, was commonly administered as a growth promoter during the livestock farming period. For this reason, health authorities are worried about how the resistance strains could spread to humans from food sources. Avoparcin received a European Union ban in 1997, which resulted in reduced VRE detection rates both in food products and farm animals. Scientific research suggests that VRE continues to survive in farm settings past the ban period because the same plasmids that harbor resistance genes vanA and ermB assist in their persistent transmission. Hospital environments in the United States primarily account for VRE occurrences because the country did not approve the use of avoparcin in animal feed products. Studies indicate that antibiotic use in livestock production creates extended health effects on AMR, which negatively influences human wellness [19].

Risk factors of VRE infections

Excessive use of vancomycin and broad-spectrum antibiotics like cephalosporins sets the conditions for VRE resistance by selecting resistant strains. The widespread use of cephalosporins and other broad-spectrum antibiotics together with cephalosporins produces VRE colonization through the destruction of normal stomach bacteria. Horizontal gene transfer via plasmids and transposons facilitates rapid resistance spread. The transmission of VRE through healthcare facilities depends on the spread of contaminated surfaces together with medical instruments and healthcare staff exposure [20]. The ICU patient population together with immunocompromised individuals and invasive device users are at high risk for VRE dissemination. The rapid dissemination of VRE happens when healthcare workers fail to practice proper infection control procedures, neglect hand hygiene rules, and do not perform adequate facility surveillance. Effective containment approaches require the implementation of strict antibiotic stewardship measures combined with infection control protocols and ongoing detection methods [20,21].

VRE vs VSE infection

The crucial goal for healthcare providers remains to distinguish VRE from vancomycin-susceptible *Enterococcus* (VSE) because the AMR influences patient results significantly. Researchers found VRE bacteremia in a hematology unit by analyzing bloodstream patterns that differed genetically from stool results thereby indicating hospital-acquired transmission beyond native intestinal population. The dissemination of intestinal microflora leads to VRE BSIs together with direct hospital transmission and gut-derived dissemination as the main infection pathways. Research indicates that immunocompromised patients demand robust infection control measures because VRE continues to spread rapidly in this setting. Patients infected with VRE experience worse outcomes than those infected with VSE because their condition leads to poor disease progression and longer hospitalization elevated healthcare expenses and increased mortality. AMR together with delayed intervention and the healthcare impacts from underlying medical conditions make VRE cases more serious. The clinical impacts of VSE infections are lower because healthcare providers can often treat these infections effectively. Resolving the growing issue of VRE demands better surveillance methods and strict adherence to infection prevention rules combined with antimicrobial stewardship practices, which work to minimize health system and patient impacts [22,23].

Vancomycin resistance

Vancomycin resistance in enterococci is mediated by the van gene operon, which can be located on a chromosome or an extrachromosomal plasmid. The resistance mechanism involves modification of the peptidoglycan precursor, replacing D-Ala-D-Ala with either D-Ala-D-Lac or D-Ala-D-Ser, reducing vancomycin binding affinity [5].

Phenotypic classification of vancomycin resistance in *Enterococcus*

Enterococci's resistance to vancomycin exists in different forms that stem from the van gene operon driving changes in peptidoglycan precursors to stop vancomycin attachment. The VanA phenotype is transferable between *E. faecium* and *E. faecalis* through plasmids or chromosomal elements yet provides cells with high resistance to both vancomycin (64-1,000 µg/mL MIC) and teicoplanin (16-512 µg/mL MIC). The VanB phenotype that occurs among *E. faecium* and *E. faecalis* demonstrates resistance to vancomycin, which follows a range from 4 to >256 µg/mL yet maintains susceptibility to teicoplanin. The resistance patterns of VanC (natural to *E. gallinarum* and *E. casseliflavus*) and the other phenotypes VanD, VanE, VanG, VanL, VanM, and VanN exhibit different degrees of transferable resistance capabilities [2-4].

Genotypic classification of vancomycin resistance in *Enterococcus*

The van gene clusters present in enterococci serve to modify the peptidoglycan precursor structure thereby decreasing the affinity of the antibiotic to its binding sites. The van operon includes vanS-vanR (functions as response regulator), vanH (gene for D-lactate dehydrogenase), vanX (gene for D-Ala-D-Ala dipeptidase), vanY (cleaves D-Ala-D-Ala pentapeptide chain), vanZ (teicoplanin resistance-determining gene, not found in vanB strains), and a variable ligase, which can be any one of the nine variant genes described to date-vanA, vanB, vanC, vanD, vanE, vanG, vanL, vanM, and vanN. These genes can be plasmid- or chromosomally encoded, with some being transposon-transferable, allowing the spreading of resistance. vanA and vanB are most clinically relevant, with vanA found on Tn1546 and vanB linked with Tn1547. vanC, vanD, vanE, vanG, vanL, vanM, and vanN have varying degrees of resistance, some being non-transferable. vanC is intrinsic to *E. gallinarum* and *E. casseliflavus*, and vanM and vanN are emerging. The vanA and vanB types of genotypes are recognized as the most widespread and important ones from a clinical perspective worldwide [10-13]. The European and Asian regions alongside Asia observe more incidents of vanA bacteria isolation as this type remains prevalent while the North American region experiences more prevalent incidents of vanB bacteria. The vanB ligase gene most commonly resides in *E. faecium* and *E. faecalis* strains, but researchers have detected cases of isolated vanA in diverse bacterial species such as *Corynebacterium* spp., *Arcanobacterium* *haemolyticum*, *Lactococcus* spp., and different *Enterococcus* species [15]. The details of different vancomycin resistance genes in *Enterococcus* are shown in Table 3.

**Table 3:** Characteristics of Vancomycin Resistance Genes in Enterococcus PEP: peptidoglycan; MIC: minimum inhibitory concentration

Gene	Expression	Location	Transferrable	PEP precursor	Vancomycin MIC (µg/mL)	Teicoplanin MIC (µg/mL)
vanA [12]	Inducible	Plasmid or chromosome	Yes	D-Ala-D-Lac	64-1,000	16-512
vanB [17]	Inducible	Plasmid or chromosome	Yes	D-Ala-D-Lac	4->256	0.5-1
vanC [18]	Inducible/constitutive	Chromosome	No	D-Ala-D-Ser	2-32	0.5-1
vanD [20]	Constitutive	Chromosome	No	D-Ala-D-Lac	64-128	4-64
vanE [14]	Inducible	Chromosome	No	D-Ala-D-Ser	16	0.5
vanG [16]	Inducible	Chromosome	Yes	D-Ala-D-Ser	8-16	0.5
vanL [21]	Inducible	Chromosome	No	D-Ala-D-Ser	8	0.5
vanM [20]	Inducible	Unknown	Yes	D-Ala-D-Lac	>256	96
vanN [22]	Constitutive	Plasmid	Yes	D-Ala-D-Ser	16	0.5

VanA-Mediated Resistance

The vanA genetic composition represents the most prevalent and strongly resistant type of VRE worldwide. The discovery of vanA in *E. faecium* first occurred on the 11-kb plasmid-borne transposon Tn1546 in 1990 before scientists found it in other *Enterococcus* species. The vanA-bearing bacteria replace D-Ala-D-Ala with D-Ala-D-Lac while both reducing glycopeptide affinity and resulting in high-level resistance to vancomycin with an MIC value of 64-1,000 μg/mL and teicoplanin with an MIC value of 16-512 μg/mL. The vanA operon exists either on plasmids or inserts into the bacterial chromosome, which enables its transfer between microorganisms leading to pan-bacterial resistance spread [11-13].

VanB-Mediated Resistance

The vanB gene provides an intermediary to strong inducible resistance to vancomycin through test results showing MIC values between 4 and >256 μg/mL and full susceptibility to teicoplanin at MIC levels between 0.5 and 1 μg/mL. The genetic entity known as vanB operon resides inside Tn5382/Tn1549-type transposons that exist both on bacterial chromosomes and plasmids and permit the transfer of genetic material between microorganisms. The genetic arrangement of the vanB operon matches the pattern of vanA except for the substitution of vanZ with vanW, which possesses an unknown function. The teicoplanin resistance is not affected due to the absence of the vanZ gene. *E. faecium* together with *E. faecalis* present the highest rates of VanB resistance in microorganisms [9-12].

Therapeutic and preventive strategies

Treatment of VRE poses difficulties because there are few available options. Medical professionals should select linezolid for both oral and intravenous administration as their first-choice antibiotic alongside daptomycin when given intravenously. Pristinamycin functions as an alternative to these two medications although tigecycline presents challenges when treating bacteremia. The treatment landscape benefits from newer antibacterial agents, which include tedizolid, telavancin, oritavancin, and dalbavancin. Methods of treatment with beta-lactams or glycopeptides together with aminoglycosides may show positive results under specific circumstances. The rise of antibiotic resistance has given rise to three alternative options including quinipristin-dalfopristin and nitrofurantoin together with fosfomycin [4-6]. The prevention of VRE depends on antibiotic surveillance programs together with controlling the misuse of vancomycin and cephalosporins along with rigorous infection control practices and environmental sanitation to prevent its transmission. The control of VRE spread relies on a comprehensive strategy that combines appropriate antibiotic limitation with infection management practices along with detection systems [1-4].

Vancomycin-dependent *Enterococcus*: a paradoxical adaptation in AMR

Mutations in the vanA or vanB operon result in the development of the VDE strain, which under normal conditions protects the bacteria from the antibiotic vancomycin. The normal VRE utilizes these operons to alter the bacteria's peptidoglycan precursors, replacing the normal D-Ala-D-Ala end with D-Ala-D-Lac, preventing vancomycin interaction and halting its activity in cell wall building [15-19]. In VDE, the bacteria produce peptidoglycan precursors only when vancomycin is present because other mutations suppress bacterial precursor production. The VDE strains with altered D-Ala-D-Lac termini bind vancomycin, but in the process, the drug itself becomes a structural stabilizer or initiates pathways that result in a resumption of cell wall building [16]. To grow, the VDE strains depend on vancomycin being present, which they can utilize but in the absence of which they perish. This antibiotic dependence illustrates how *Enterococcus* species went on to change their functions and further complicates diagnosis and treatment [19].

## Conclusions

Scientists previously considered *Enterococcus* species as harmless microorganisms until they became major hospital-based infections that trigger multiple healthcare-related infections. The antibiotic-resistant nature combined with intrinsic resistance and exceptional multidrug resistance abilities of *Enterococcus* species presents strong challenges to treatment, especially during the growing rise of VRE infections. These pathogens demonstrate clinical importance through their capability to survive in hospital environments as well as their ability to colonize medical devices and their ability to overcome immune responses. Worldwide VRE rates continue rising because broad-spectrum antibiotics like cephalosporins are getting overused and also because of the horizontal transfer of resistance genes. Multiple techniques must be combined for effective control, which involves robust infection prevention measures as well as antimicrobial management and constant monitoring systems. The chronic resistance crisis emphasizes the necessity of developing fresh antimicrobial strategies along with different treatment alternatives to oppose hard-to-treat and resilient pathogens.

## References

[1] Doss Susai Backiam A, Duraisamy S, Karuppaiya P, Balakrishnan S, Chandrasekaran B, Kumarasamy A, Raju A (2023). Antibiotic susceptibility patterns and virulence-associated factors of vancomycin-resistant enterococcal isolates from tertiary care hospitals. Antibiotics (Basel).

[2] Rao C, Dhawan B, Vishnubhatla S, Kapil A, Das B, Sood S (2021). Clinical and molecular epidemiology of vancomycin-resistant Enterococcus faecium bacteremia from an Indian tertiary hospital. Eur J Clin Microbiol Infect Dis.

[3] Kankalil George S, Suseela MR, El Safi S (2021). Molecular determination of van genes among clinical isolates of enterococci at a hospital setting. Saudi J Biol Sci.

[4] Das AK, Dudeja M, Kohli S, Ray P, Singh M, Kaur PS (2020). Biofilm synthesis and other virulence factors in multidrug-resistant uropathogenic enterococci isolated in Northern India. Indian J Med Microbiol.

[5] Das AK, Dudeja M, Kohli S, Ray P (2022). Genotypic characterization of vancomycin-resistant Enterococcus causing urinary tract infection in northern India. Indian J Med Res.

[6] Mohanty S, Behera B (2022). Antibiogram pattern and virulence trait characterization of Enterococcus species clinical isolates in Eastern India: a recent analysis. J Lab Physicians.

[7] Sachan S, Anubhaw A (2022). Species prevalence, antimicrobial susceptibility and detection of virulence factors of enterococci isolated from tertiary care hospital. Int J Health Sci.

[8] CLSI CLSI (2025). CLSI M100 Performance Standards for Antimicrobial Susceptibility Testing, 35th Edition. Performance Standards for Antimicrobial Susceptibility Testing.

[9] Saengsuwan P, Singkhamanan K, Madla S, Ingviya N, Romyasamit C (2021). Molecular epidemiology of vancomycin-resistant Enterococcus faecium clinical isolates in a tertiary care hospital in southern Thailand: a retrospective study. PeerJ.

[10] Sannathimmappa MB, Nambiar V, Aravindakshan R, Al-Risi ES (2023). Clinical profile and antibiotic susceptibility pattern of Enterococcus faecalis and Enterococcus faecium with an emphasis on vancomycin resistance. Biomed Biotechnol Res J.

[11] Elaskary SA, Zaher EM (2022). Linezolid susceptibility and virulence factors in vancomycin resistant Enterococcus faecalis and Enterococcus faecium among hospitalized burn patients. Egypt J Med Microbiol.

[12] Haghi F, Lohrasbi V, Zeighami H (2019). High incidence of virulence determinants, aminoglycoside and vancomycin resistance in enterococci isolated from hospitalized patients in Northwest Iran. BMC Infect Dis.

[13] Kateete DP, Edolu M, Kigozi E, Kisukye J, Baluku H, Mwiine FN, Najjuka CF (2019). Species, antibiotic susceptibility profiles and van gene frequencies among enterococci isolated from patients at Mulago National Referral Hospital in Kampala, Uganda. BMC Infect Dis.

[14] Shah AA, Khursheed S, Rashid A, Gupta A (2022). Isolation, identification, speciation, and antibiogram of Enterococcus species by conventional methods and assessment of the prevalence of vana genotype among VRE. J Med Pharm Allied Sci.

[15] Georges M, Odoyo E, Matano D (2022). Determination of Enterococcus faecalis and Enterococcus faecium antimicrobial resistance and virulence factors and their association with clinical and demographic factors in Kenya. J Pathog.

[16] Farman M, Yasir M, Al-Hindi RR, Farraj SA, Jiman-Fatani AA, Alawi M, Azhar EI (2019). Genomic analysis of multidrug-resistant clinical Enterococcus faecalis isolates for antimicrobial resistance genes and virulence factors from the western region of Saudi Arabia. Antimicrob Resist Infect Control.

[17] Ahmed J, Yadav RK, Sood S, Das BK, Dhawan B (2023). Vancomycin-resistant Enterococcus faecium: a high priority pathogen. J Appl Sci Clin Pract.

[18] Saengsuwan P, Singkhamanan K, Kawila S, Romyasamit C (2023). Vancomycin-resistant enterococci isolated in tertiary care in Southern Thailand: prevalence and characterization of biofilm formation. Rev Rom Med Lab.

[19] Torres C, Alonso CA, Ruiz‐Ripa L, León-Sampedro R, del Campo R, Coque TM (2018). Antimicrobial resistance in Enterococcus spp. of animal origin. Antimicrobial Resistance in Bacteria From Livestock and Companion Animals.

[20] Fahmy N, Abdel-Gawad AR, Rezk GA, Mahmoud EA (2021). Characterization of Enterococci isolated from intensive care unit (ICU); distribution of virulence markers, virulence genes and antibiotic resistance pattern. Microbes Infect Dis.

[21] Orak F, Yalçınkaya KT, Aydın F (2024). Determination of glycopeptide resistance genes and virulence factors in vancomycin-resistant enterococci isolates and the relationship between glycopeptide resistance genes and endogenous/exogenous flora. J Clin Pract Res.

[22] Geraldes C, Tavares L, Gil S, Oliveira M (2022). Enterococcus virulence and resistant traits associated with its permanence in the hospital environment. Antibiotics (Basel).

[23] Jakovac S, Bojić EF, Ibrišimović MA, Tutiš B, Ostojić M, Hukić M (2017). Characteristics of vancomycin-resistant Enterococcus strains in the West Balkans: a first report. Microb Drug Resist.

